# Clinical significance of FBXW7 tumor suppressor gene mutations and expression in human colorectal cancer: a systemic review and meta-analysis

**DOI:** 10.1186/s12885-021-08535-8

**Published:** 2021-07-03

**Authors:** Wei Shang, Chuanwang Yan, Ran Liu, Lili Chen, Dongdong Cheng, Liang Hao, Wenguang Yuan, Jingbo Chen, Hui Yang

**Affiliations:** 1 Department of General Surgery, Key Laboratory of Metabolism and Gastrointestinal Tumor, Key Laboratory of Laparoscopic Technology, Shandong Medicine and Health Key Laboratory of General Surgery, The First Affiliated Hospital of Shandong First Medical University & Shandong Provincial Qianfoshan Hospital, the First Affiliated Hospital of Shandong First Medical University, Jinan, 250000 Shandong China; 2 Department of General Surgery, Shandong Provincial Qianfoshan Hospital, Weifang Medical College, Weifang, 261000 Shandong China; 3 Department of Pathology, Jinan Central Hospital, Jinan, 250000 Shandong China; 4 Department of General Surgery, Feicheng Hospital of Shandong Guoxin Yiyang Group, Tai’an, 271600 Shandong China; 5 Department of Gastrointestinal Surgery, Zibo First People’s Hospital, Zibo, 255000 Shandong China

**Keywords:** FBXW7, Mutation, Expression, Survival, Cancer

## Abstract

**Background:**

Various studies investigating the clinical significance of FBXW7 mutation and/or expression have yielded inconclusive results in colorectal cancer (CRC) patients. Therefore, the present meta-analysis summarizes previous evidence and evaluates the clinical significance, including the prognostic role, of FBXW7 status in CRCs.

**Methods:**

The meta-analysis was conducted by searching the databases of PubMed, China National Knowledge Infrastructure (CNKI), WANFANG data, Web of Science, Embase, and Web of Science. Pooled odds ratios (ORs) and hazard ratios (HRs) and corresponding 95% confidence intervals (CIs) were calculated to assess the relationships between FBXW7 status and clinicopathological features and survival in CRC, respectively.

**Results:**

Ten studies involving 4199 patients met the inclusion criteria and included in our meta-analysis. FBXW7 mutation/low expression was obviously correlated with advanced T stage (OR = 0.44, 95% CI: 0.27–0.74, *P* <  0.01) and lymph node metastasis (OR = 1.88, 95% CI: 1.40–2.53, *P* <  0.01), but was not associated with other parameters. Further investigation found that FBXW7 mutation/low expression predicted poor OS (HR = 1.25, 95% CI: 1.06–1.47, *P* <  0.01), but not DFS in CRC (HR = 1.04, 95% CI: 0.60–1.82, *P* = 0.88). Subgroup analysis found that FBXW7 status was obviously correlated with OS in cohorts recruited after 2009 (HR = 1.32, 95% CI: 1.17–1.50, *P* <  0.01), from eastern Asia (HR = 1.27, 95% CI: 1.04–1.55, *P* = 0.02), detected by immunohistochemistry/qRT-PCR (HR = 1.39, 95% CI: 1.22–1.59, *P* <  0.01), and analysed with multivariate method (HR = 1.47, 95% CI: 1.25–1.74, *P* <  0.01).

**Conclusions:**

This study indicates that FBXW7 status, expression level especially, is associated with OS but not DFS in CRC. FBXW7 expression level may function as a prognostic biomarker in CRC.

## Background

Colorectal cancer (CRC) ranks the forth most commonly diagnosed cancer and the second leading cause of cancer-related death worldwide [1]. Based on the most recent data, the annual age standardized CRC incidence rate was 38.7 per 100,000 persons (2012–2016), and the mortality rate was 13.9 per 100,000 persons (2013–2017) [2]. Despite recent advances in therapy and multidisciplinary care in CRC, about 900,000 individuals die from this malignancy [3]. Fortunately, recent advances in genomic sequencing and molecular based cancer development pathways now allow for a deeper understanding of pathogenesis [4]. Some well-known genes in CRC may provide opportunities for targeted clinical interventions or/and survival prediction.

FBWX7 (F-box and WD repeat domain-containing 7) is the substrate recognition component of an evolutionary conserved SCF (complex of SKP1, CUL1 and F-box protein)-type ubiquitin ligase [5]. Functioning as a general tumor suppressor in human cancer, FBXW7 is the most frequently mutated of SCF-type ubiquitin ligase in human cancer cells [6]. Besides, it has been shown to degrade several proto-oncogenes that function in cellular growth and division pathways, including cyclin E1, c-Myc, c-Jun, and Notch [7]. The altered status of FBXW7 is recognized to be one of the major causes of carcinogenesis or cancer development [5, 7, 8]. CRC harbors the second most frequent FBXW7 mutations (7.73%) among different cancer types [9]. Moreover, FBXW7 is one of the most frequently mutated genes during CRC initiation and progression [10]. Altered FBXW7 status (mutation and/or low expression) may be associated with prognosis in CRC, however, the results vary among different studies [11–20]. Thus, we conducted a systematic review and meta-analysis of data from previous studies to quantitatively assess the association between FBXW7 status and survival in CRC.

## Methods

### Literature search and study selection

**Fig. 1:**
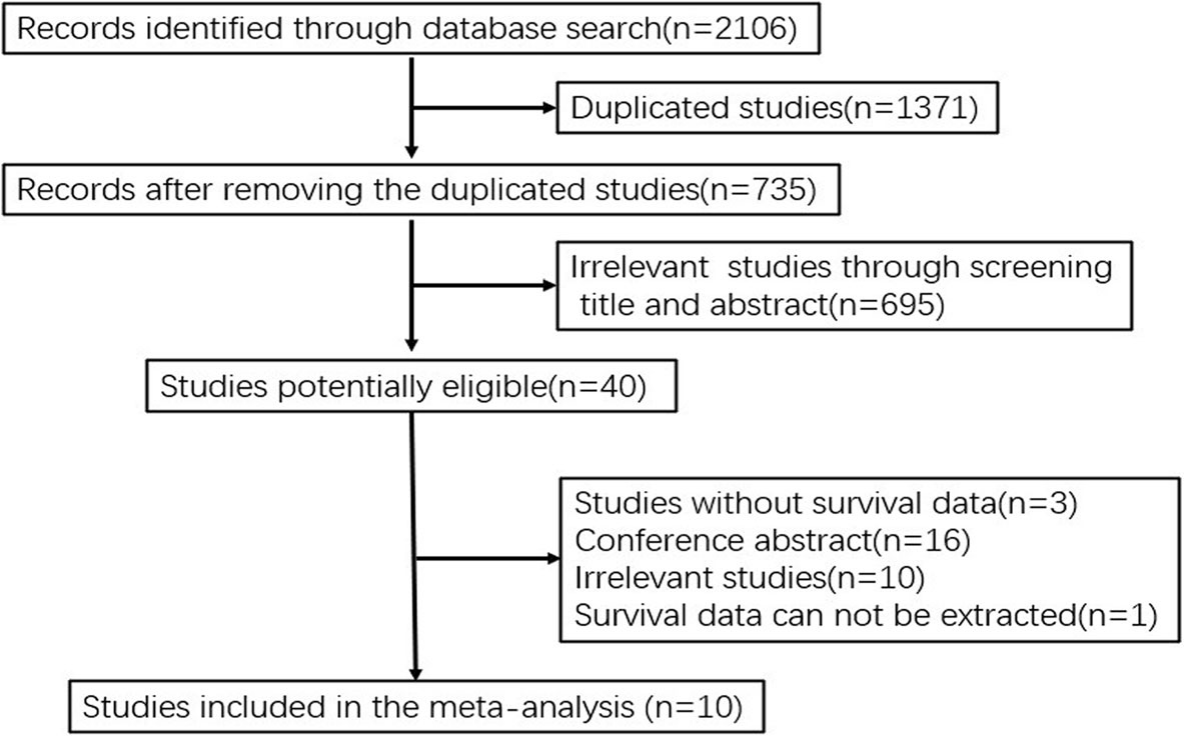
A flow chart of this study

A systematic literature search of PubMed, China National Knowledge Infrastructure (CNKI), WANFANG data, Web of Science, Embase, and Web of Science was performed in September, 2020. The following key words or text words were used: “FBXW7”, “CDC4”, “CRC”, “colon”, “rectum”, “intestinal”, “cancer”, “carcinoma”, “tumor”, “prognosis”, “survival”. Eligible articles should meet the following criteria: (1) CRC was pathologically confirmed; (2) studies investigated the association of FBXW7 mutation and/or expression with survival outcome; (3) the hazard ratio (HR) and 95% confidence interval (CI) for survival were provided or could be calculated from the available data. Articles were excluded based on any of the following criteria: (1) studies lacking essential information for calculating HR and 95% CI; (2) reviews, comments, letters, case reports, and conference abstracts; (3) neither English nor Chinese articles. When multiple publications of a study were identified, the most detailed version for meta-analysis was selected. A flow diagram of the study selection process is presented in Fig. 1.

### Data extraction

Two reviewers (WS and CWY) independently extracted the following data from each study: basic study information (name of first author, year of publication, region or country where the study was conducted, number of patients, follow-up period, and analysis method of survival), participant characteristics (age and gender), FBXW7 related data (detection method, cutoff score, antibody source and dilution, the HRs of FBXW7 mutation/expression for overall survival (OS), disease-free survival (DFS), as well as their 95% CIs and *P* values) and clinical parameters (histological type, tumor size, tumor location, venous invasion, peritoneal metastasis, lymph node metastasis, distant metastasis, TNM stage and Duke’s stage). If available, HRs and 95% CIs were preferentially obtained from multivariate results. Otherwise, they were extracted from univariable outcomes or calculated using Engauge Digitizer version 4.1 (free software down-loaded from http://sourceforge.net) to read the Kaplane-Meier survival curves to get the HRs and 95% CIs [21–23]. Discrepancies were adjudicated by a third reviewer (RL) until a consensus was reached.

### Quality assessment

The quality of all eligible studies were assessed independently by 2 investigators (CWY and RL) using the Newcastle-Ottawa quality assessment scale (NOS). All disagreements were discussed and resolved with consensus. The NOS criteria was scored based on three aspects: (1) subject selection, (2) comparability of subject, (3) clinical outcome. Scores based on NOS of 7–9 indicate a good-quality study, scores of 4–6 indicate an intermediate-quality study, and scores less than 4 indicate a low-quality study.

### Statistical analysis

Statistical analysis was performed using Stata statistical software version 12.0 (Stata Corporation, College Station, Texas, USA) and Review Manager version 5 (RevMan; The Nordic Cochrane Centre, Copenhagen, Denmark). Odds ratios (ORs) with 95% confidence intervals (CIs) were estimated to evaluate the association between FXBW7 status and the clinicopathological features in CRC. The statistical significance of the pooled OR and HR was evaluated with the Z test and *P* values, and *P* <  0.05 was considered statistically significant. Subgroup analysis was conducted to determine the source of existing heterogeneity. Heterogeneity among studies was determined by employing the Q and I^2^ statistics. If the 푃 value was greater than 0.1 and the 퐼^2^-value was less than 50%, the heterogeneity among studies did not reach statistical significance, and the fixed-effects model was subsequently implemented. Otherwise, the random-effects model was used. Publication bias was assessed by the Begg’s rank correlation method and Egger’s weighted regression method, and a *P* value less than 0.05 was considered statistically significant. In addition, a sensitivity analysis was performed to assess the influence of a single study on pooled HR.

## Results

### Study selection and description of the include studies

A total of 2106 articles were obtained through database search. After removing duplicated studies and irrelevant studies through screening title and abstract, 40 studies were remained. Then, the full texts of the articles were reviewed in detail, and 10 studies met our inclusion criteria were finally included for the meta-analysis, including 4 studies detecting FBXW7 mutation and 6 studies measuring FBXW7 expression. The main characteristics of the included studies are presented in Table 1. These studies were published between 2009 and 2019, and conducted in 4 countries (China, Australia, America, and Japan). The overall sample size was 4199, ranging from 50 to 1519. The relationship between OS and FXBW7 status was all described in the 10 studies, and DFS was reported in 4 studies. All of the eligible entries scored more than five by NOS, revealing a high methodological quality across all studies. FBXW7 expression was measured by IHC or qRT-PCR, and mutation was detected though different sequencing methods. For the purposes of this anlaysis, cases with low expression of FBXW7 or coding mutations were considered one similar group of patients that had tumors with a deficit in FBXW7.

**Table 1:** Characteristics of studies included in the present meta-analysis

Detection of FBXW7 mutation/expression	Study	Study Region	Recruitment time	No. of patients	Clinical Stage	FBXW7 status method	Cut off	Antibody source	Dilution	Case: Low/High (MT/WT)^**a**^	Median follow-up months	Analysis method	OS HR(95%CI)	DFS HR(95%CI)	Quality score
Mutation	Chang, 2015 [11]	Taiwan	2000-2009	1519	TNM I-IV	MassArray	NA	NA	NA	114/1405	NA	Univariate	1.00 (0.98–1.02)	NA	8
	Mouradov, 2013 [19]	Australia	2002-2004	822	TNM II-III	Sanger sequencing	NA	NA	NA	41/781	32.2	Univariate	0.96 (0.45–2.06)	NA	7
	Korphaisarn, 2017 [16]	USA	2009-2015	527	TNM IV	NGS	NA	NA	NA	43/484	30.4	Multivariate	2.00 (1.27–3.16)	NA	8
	Iwatsuki, 2010 [14]	Japan	1993-1999	93	Duke A-D	qRT-PCR	Median	NA	NA	46/47	36	Multivariate	1.98 (1.26–3.27)	NA	7
	Gao, 2019 [12]	China	2015-2016	207	TNM I-IV	MiSeq	NA	NA	NA	33/174	23	Univariate	0.59 (0.21–1.68)	0.75 (0.32–1.79)	7
Expression	He, 2019 [13]	China	2009-2011	140	TNM I-IV	IHC	NA	Abcam, USA	1:500	84/56	NA	Univariate	2.30 (0.92–5.76)	2.45 (1.22–4.92)	6
	Liu, 2018 [18]	China	2010-2015	509	TNM I-IV	IHC	Score 4	Abcam, USA	1:200	359/150	NA	Univariate	2.22 (1.40–3.45)	NA	6
	Li, 2018 [17]	China	2007-2009	276	TNM I-IV	IHC	Score 1	Bethyl, USA	NA	60/216	NA	Multivariate	3.57 (2.23–5.71)	4.63 (2.65–8.13)	7
	Tang, 2016 [24]	China	2011-2011	50	Duke A-D	IHC	score 3	Santa Cruz, USA	1:60	23/27	NA	Univariate	1.04 (0.12–9.42)	NA	7
	Kawashita, 2017 [15]	Japan	2001-2009	56	NA	IHC	Score 3	Abcam, USA	1:100	24/32	55	Univariate	1.98 (0.42–9.26)	1.50 (0.79–2.85)	7

^a^ Low/High indicates low expression of FBXW7 versus high expression of FBXW7 in studies investigating the RNA or protein level of FBXW7, and the MT/WT implies the mutation of FBXW7 versus wild type of FBXW7 in studies investigating the mutation rate of FBXW7

### Correlation between FBXW7 and clinicopathological features

Correlation between FBXW7 status and clinicopathological features was presented in 8 studies. Based on the ORs derived from these studies, we evaluated the correlation between FBXW7 status and some clinicopathological characteristics, including age, gender, histological grade, tumor size, tumor location, venous invasion, peritoneal metastasis, depth of invasion, lymph node metastasis, distant metastasis, TNM stage and Duke’s stage. (Table 2) Aberrant FBXW7 status was significantly associated with advanced T stage (OR = 0.44, 95% CI: 0.27–0.74, *P* <  0.01) and lymph node metastasis (OR = 1.88, 95% CI: 1.40–2.53, *P* <  0.01). Frequency of venous invasion was also higher in FBXW7 mutation/low expression cohort, but no statistical significance was detected (OR = 1.63, 95% CI: 1.01–2.64, *P* = 0.05). No obvious relationship was verified between FBXW7 status and other parameters. (Table 2).

**Table 2:** Meta-analysis of FXBW7 status and clinicopathological features in CRC

Parameters Characteristics	Number of studies	OR (95%CI)	I^2^ (%)	*P*_h_	Z	*P* value
Age(≥ 60 year vs. < 60 year)	3	1.00 (0.93–1.36)	0	0.71	0.00	1.00
Gender (Male vs. Female)	7	1.03 (0.83–1.28)	6	0.38	0.28	0.78
Differentiation(Well vs. Moderate + Poor)	2	0.81 (0.40–1.64)	0	0.63	0.89	0.37
Differentiation(Well+ Moderate vs. Poor)	4	0.72 (0.35–1.48)	69	0.02	0.59	0.55
Size(≥ 5 cm vs. < 5 cm)	3	0.93 (0.64–1.35)	0	0.45	0.37	0.71
Tumor location(Colon vs. Rectum)	5	0.85 (0.64–1.12)	30	0.22	1.17	0.24
Venous invasion(Present vs. Absent)	3	1.63 (1.01–2.64)	14	0.31	1.99	0.05
Peritoneal metastasis (Present vs. Absent)	2	0.82 (0.38–1.80)	0	0.40	0.49	0.63
Depth of invasion (T1 + T2 vs. T3 + T4)	3	0.44 (0.27–0.74)	0	0.99	3.12	< 0.01
Lymph node metastasis (Positive vs. Negative)	5	1.88 (1.40–2.53)	0	0.45	4.18	< 0.01
Distant metastasis (Present vs. Absent)	3	1.85 (0.34–10.24)	92	< 0.01	0.71	0.48
TNM stage(I + II vs. III + IV)	3	0.53 (0.15–1.84)	95	< 0.01	1.00	0.32
Duke’s stage(A + B vs. C + D)	2	0.45 (0.04–5.20)	90	< 0.01	0.64	0.52

### Prognostic value of FBXW7

**Fig. 2:**
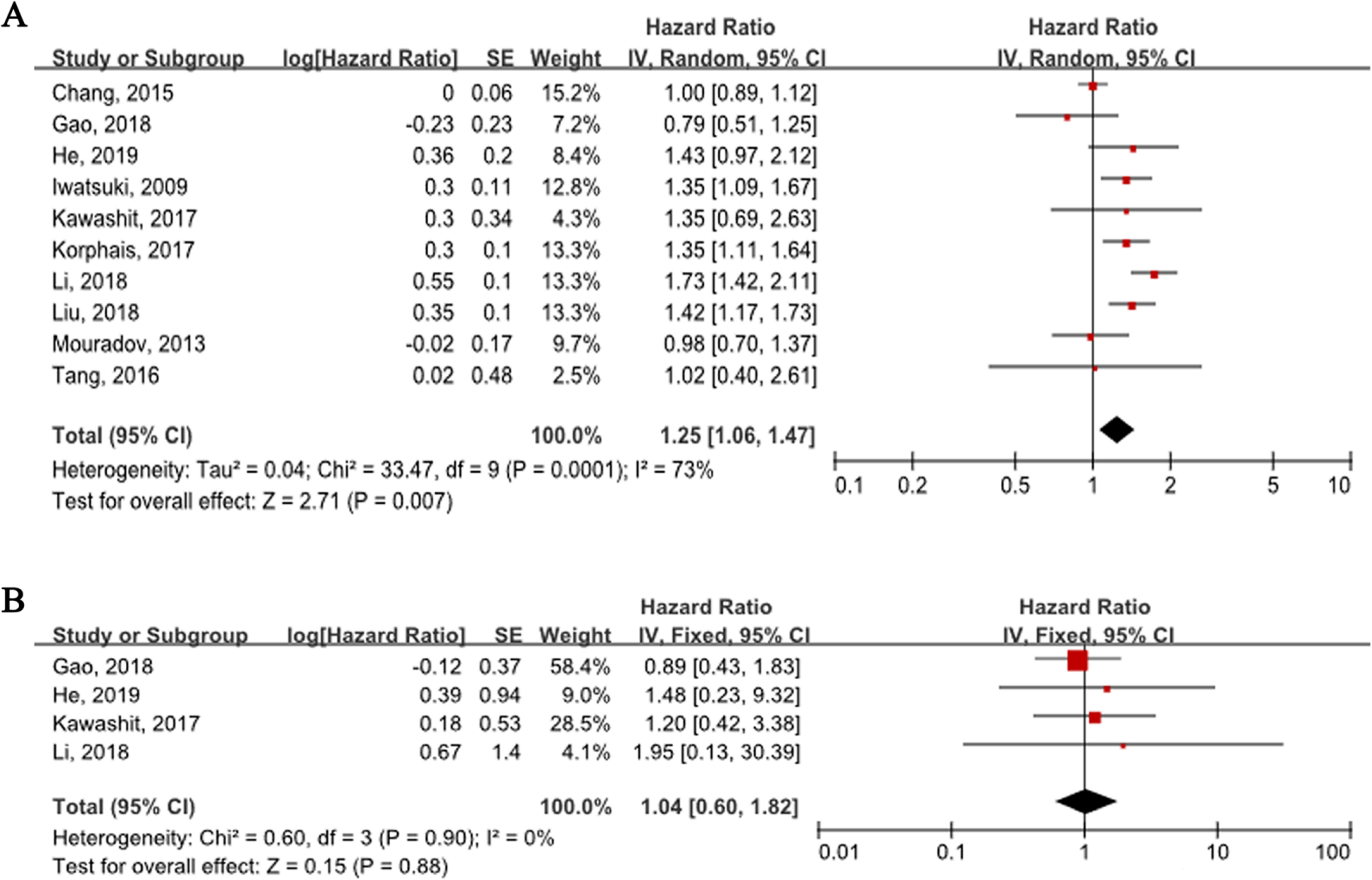
Forest plots: Summary hazard ratios (HRs) and 95% confidence intervals (CIs) of colorectal cancer OS (**a**) and DFS (**b**) for FBXW7 status

All the 10 studies were enrolled to detect the prognostic value of FBXW7 in OS. A random-effect model was used to calculate the pooled HR and 95% CI because excessive heterogeneity existed between studies (*P* <  0.01, I^2^ = 73%). (Fig. 2a) Overall, FBXW7 mutation/low expression predicted poor OS (HR = 1.25, 95% CI: 1.06–1.47, *P* <  0.01). (Fig. 2a) However, no significant correlation was found between FBXW7 and DFS in CRC (HR = 1.04, 95% CI: 0.60–1.82, *P* = 0.88). (Fig. 2b) To detect potential heterogeneity, subgroup analyses were stratified based on recruitment time, region, FBXW7 detection method, sample size and data type to evaluate FXBW7 prognostic value in CRC. As shown in Table 3, FBXW7 mutation/low expression predicted decreased OS regardless of sample size ≥100 (HR = 1.23, 95% CI: 1.01–1.51, *P* = 0.04) or <  100 (HR = 1.33, 95% CI: 1.09–1.63, *P* <  0.01). Besides, FBXW7 status was obviously correlated with OS in cohorts recruited after 2009 (HR = 1.32, 95% CI: 1.17–1.50, *P* <  0.01), from eastern Asia (HR = 1.27, 95% CI: 1.04–1.55, *P* = 0.02), detected by IHC/qRT-PCR (HR = 1.39, 95% CI: 1.22–1.59, *P* <  0.01), and analysed with multivariate method (HR = 1.47, 95% CI: 1.25–1.74, *P* <  0.01). However, no prognostic effect was observed in patients recruited before 2009 (HR = 1.24, 95% CI: 0.93–1.65, *P* = 0.14), from regions beyond eastern Asia (HR = 1.18, 95% CI: 0.87–1.61, *P* = 0.28), detected by sequencing (HR = 1.17, 95% CI: 0.94–1.47, *P* = 0.16), and analysed with univariate method (HR = 1.13, 95% CI: 0.94–1.35, *P* = 0.20). (Table 3).

**Table 3:** Subgroup analyses for overall survival

Outcome	Characteristics	Number of studies	HR(95%CI)	I^2^ (%)	P_h_	Z	*P* value
Recruitment time	Before 2009	5	1.24 (0.93–1.65)	88	< 0.01	1.46	0.14
	After 2009	5	1.32 (1.17–1.50)	32	0.21	4.43	< 0.01
Region	Eastern asia	8	1.27 (1.04–1.55)	77	< 0.01	2.31	0.02
	Other regions	2	1.18 (0.87–1.61)	62	0.10	1.08	0.28
FBXW7 detection method	IHC/qRT-PCR	6	1.39 (1.22–1.59)	46	0.10	4.95	< 0.01
	Sequencing	4	1.17 (0.94–1.47)	73	0.01	1.40	0.16
Sample Size	≥ 100	7	1.23 (1.01–1.51)	81	< 0.01	2.06	0.04
	< 100	3	1.33 (1.09–1.63)	0	0.85	2.81	< 0.01
Data types	Univariate	7	1.13 (0.94–1.35)	56	0.03	1.28	0.20
	Multivariate	3	1.47 (1.25–1.74)	50	0.13	4.56	< 0.01

### Publication bias and sensitivity analysis

**Fig. 3:**
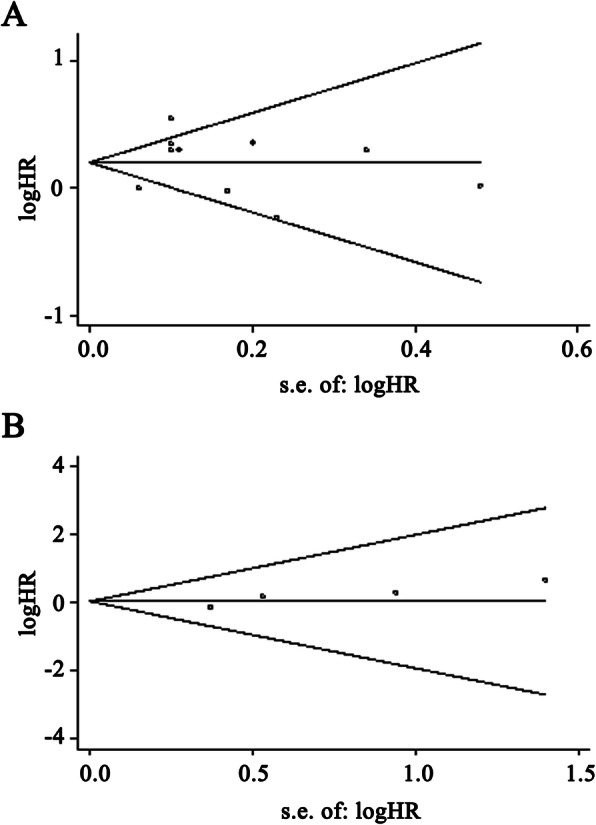
Begg’s funnel plots of the natural logarithm of the hazard ratios (HRs) and the SE of the natural logarithm of the HRs for the included studies reported with OS (**a**) and PFS (**b**)

A funnel plot, with regard to the publication bias of all studies for OS and four studies for DFS, showed the basic symmetrical. (Fig. 3a and b) Evaluation of publication bias using Begg’s and Egger’s tests also showed that no publication bias existed (*P* value of Begg’s test, 0.24 and 0.31 for OS and DFS, respectively; *P* value of Egger’s test, 0.75 and 0.08 for OS and DFS, respectively). Furthermore, to evaluate the results of meta-analysis, sensitivity analysis was conducted. No significant change was found in the results when any 1 study was excluded, confirming the robustness and reliability of meta-analysis results on both OS and DFS (Table 4).

**Table 4:** The influence of individual study on the pooled estimate for outcomes

Outcome	Study omitted	HR(95%CI)	I^2^ (%)	*P*_h_	Z	*P* value
OS	Chang, 2015 [11]	1.32 (1.15–1.52)	49	0.05	3.93	< 0.01
	He, 2019 [13]	1.23 (1.04–1.47)	76	< 0.01	2.38	0.02
	Mouradov, 2013 [19]	1.28 (1.08–1.52)	75	< 0.01	2.83	< 0.01
	Liu, 2018 [18]	1.22 (1.02–1.47)	74	< 0.01	2.18	0.03
	Korphaisarn, 2017 [16]	1.23 (1.02–1.48)	75	< 0.01	2.21	0.03
	Iwatsuki, 2010 [14]	1.19 (1.03–1.38)	59	0.01	2.39	0.02
	Li, 2018 [17]	1.23 (1.03–1.48)	75	< 0.01	2.24	0.03
	Tang, 2016 [24]	1.26 (1.06–1.48)	76	< 0.01	2.69	< 0.01
	Gao, 2019 [12]	1.29 (1.10–1.52)	73	< 0.01	3.10	< 0.01
	Kawashita, 2017 [15]	1.24 (1.05–1.47)	76	< 0.01	2.55	0.01
DFS	He, 2019 [13]	1.01 (0.56–1.80)	0	0.80	0.03	0.97
	Li, 2018 [17]	1.02 (0.58–1.79)	0	0.82	0.06	0.95
	Gao, 2018 [12]	1.31 (0.56–3.11)	0	0.94	0.62	0.53
	Kawashita, 2017 [15]	0.99 (0.51–1.91)	0	0.78	0.03	0.98

## Discussion

Our team has focused on investigating the functional role of FBXW7 in multiple cancers, including in CRC [25–28]. FBXW7 is one of most frequently mutated and downregualted genes in CRC, however, the clinical significance and prognostic value of FBXW7 in CRC have not been specified. To our known, this is the first meta-analysis to provide comprehensive evidence of the association between FBXW7 status and prognosis in CRC. Mutation in this study indicated all mutations whether accompanied with loss of function or not. Mutation detection has some advantages, for example, the mutation of FBXW7 can be detected in patients not underwent operation and cancer tissues can not be achieved, which are necessary for protein detecting. Pooled data of 4199 CRC patients confirmed that FBXW7 mutation and expression loss were detected in 7.5 and 53.0% cases, respectively. Previous study has indicated that FBXW7 could repress the migratory and invasive capacities of CRC cells through inhibiting stem cell-like behavior and epithelial-mesenchymal transition [26]. This meta-analysis suggests that FBXW7 mutation and/or low expression was significantly associated with advanced T stage and lymph node metastasis. Venous invasion rate was also higher in FBXW7 mutation/low expression cohort, though not statistically significant. This evidence indicates the essential role of FBXW7 in local invasion and metastasis in CRC. In addition, FBXW7 missense mutations have been shown to have a strong negative prognostic association in CRC [16], the association between FBXW7 status and distant metastasis was not discovered in our meta-analysis (OR = 1.85, 95% CI: 0.34–10.24, *P* = 0.48). Moreover, tumor size and clinical stage were not correlated with FBXW7 status as revealed in this study. Taken together, FBXW7 may influence the survival outcomes of CRC patients through regulating local invasion and lymph node metastasis but not tumor growth.

Our meta-analysis found that FBXW7 mutation/low expression predicted poor OS, but not DFS in CRC. When subgroup analysis was conducted, FBXW7 status was correlated with OS in cohorts analysis with multivariate method, but not with univariate method. Results from multivariate analysis, which took other clinicopathological parameters into consideration simultaneously, are more accurate than univariate analysis. Besides, FBXW7 mRNA/protein level was correlated with OS, but FBXW7 mutation alone was not. Previous study has found that FBXW7 mutations are not predicted to cause loss of function [29]. FBXW7 mRNA/protein level may be more valuable in predicating prognosis. As reported previously, FBXW7 mutated CRC patients resistant to anti-epidermal growth factor receptor (EGFR) immunotherapy treatment (monoclonal antibodies, Cetuximab or Panitumumab) [30]. Besides, loss of FBXW7 is associated with drug resistance to Oxaliplatin [31]. It has been reported that rapamycin could inhibit FBXW7 loss-induced epithelial-mesenchymal transition and cancer stem cell-like characteristics in CRC cells [6, 26]. And rapamycin could inhibit tumor metastasis in vivo in cholangiocarcinoma [27]. However, application of FBXW7 signaling pathway targeted therapies in human is no clue yet.

This meta-analysis has several limitations to be discussed. First, significant heterogeneity was observed among the included studies. By excluding each study individually, sensitivity analysis revealed that the predictive significance of FBXW7 status on OS in CRC. Second, there was some unavoidable variability in study designs, such as the sequence method or antibody used for FBXW7 mutation or expression detection, TNM stage of the involved patients and the cutoff value for dichotomizing FBXW7 low or high expression. And the variability among studies is indeed a problem when determining the significance of FBXW7. Fortunately, publication bias was not detected for all the studies for OS and four studies for DFS, and sensitivity analysis revealed that no significant change was found in the results when any 1 study was excluded. Third, several studies having small numbers of patients recruited. Finally, publication bias may be a problem in meta-analyses though not detected using Begg’s and Egger’s tests. All relevant data were tired to identified, and additional unpublished information was retrieved, but some missing data were unavoidable.

## Conclusions

Altered FBXW7 status was associated with advanced T stage and lymph node metastasis in CRC, and low FBXW7 mRNA/protein level indicates poor OS in CRC. FBXW7 may be a potential prognostic biomarker in CRC patients. These findings may provide evidence for determining therapeutic regimen in CRC patients.

## Data Availability

The datasets used and/or analyzed during the current study are available from the corresponding author on reasonable request.
